# The Role of Public Health in Addressing Racial and Ethnic Disparities in Mental Health and Mental Illness

**Published:** 2009-12-15

**Authors:** Annelle B. Primm, Melba J. T. Vasquez, Robert A. Mays, Doreleena Sammons-Posey, Lela R. McKnight-Eily, Letitia R. Presley-Cantrell, Lisa C. McGuire, Daniel P. Chapman, Geraldine S. Perry

**Affiliations:** Minority and National Affairs, American Psychiatric Association; American Psychological Association, Washington, DC; National Institute of Mental Health, Bethesda, Maryland; National Association of Chronic Disease Directors and Directors of Health Promotion and Education, Trenton, New Jersey; Centers for Disease Control and Prevention, Atlanta, Georgia; Centers for Disease Control and Prevention, Atlanta, Georgia; Centers for Disease Control and Prevention, Atlanta, Georgia; Centers for Disease Control and Prevention, Atlanta, Georgia; Centers for Disease Control and Prevention, Atlanta, Georgia

## Abstract

Racial/ethnic minority populations are underserved in the American mental health care system. Disparity in treatment between whites and African Americans has increased substantially since the 1990s. Racial/ethnic minorities may be disproportionately affected by limited English proficiency, remote geographic settings, stigma, fragmented services, cost, comorbidity of mental illness and chronic diseases, cultural understanding of health care services, and incarceration. We present a model that illustrates how social determinants of health, interventions, and outcomes interact to affect mental health and mental illness. Public health approaches to these concerns include preventive strategies and federal agency collaborations that optimize the resilience of racial/ethnic minorities. We recommend strategies such as enhanced surveillance, research, evidence-based practice, and public policies that set standards for tracking and reducing disparities.

## Introduction

Racial and ethnic diversity is increasing in the United States; by 2042, racial/ethnic minorities are expected to surpass non-Hispanic whites as the majority population in the United States (1). Nevertheless, racial/ethnic minority populations remain underserved in the mental health care system (2). The Surgeon General advocates a public health approach to eliminate disparities in mental health among racial/ethnic minorities (3). The New Freedom Commission on Mental Health recommends providing access to high-quality and culturally effective care that includes underserved, remote, and rural areas of the country and recommends that states monitor mental health service disparities through their comprehensive state mental health plans (2).

We describe mental health disparities among racial/ethnic minority populations, such as differences in prevalence rates, diagnoses, access to care, and sources of care. We propose a model that illustrates the relationships between the social determinants of mental health, the necessary public health interventions, and the expected positive outcomes resulting from these interventions in racial/ethnic minority populations. Finally, we offer additional public health recommendations for consideration.

## Existing Mental Health Disparities in Racial/Ethnic Minority Populations

Health disparities are defined as "differences in the overall rate of disease incidence, prevalence, morbidity, mortality, or survival" (4). The Surgeon General's report on mental health noted that mental health and mental illness are not polar opposites (5). Mental health is the successful performance of mental function, resulting in productive activities, fulfilling relationships, and the ability to adapt to change and adversity. Mental illness refers to mental disorders characterized by alterations in thinking, mood, or behavior associated with distress or impaired functioning. The evolution and nuances of definitions of mental health and mental illness are discussed in depth elsewhere in this issue of *Preventing Chronic Disease* (6).

The prevalence of mental illness differs between whites and racial/ethnic minority populations. For example, among American Indian tribes, the lifetime prevalence of any psychiatric disorder is higher (50%-54% for men, 41%-46% for women) than among the overall US population (44% for men, 38% for women) (7). The prevalence of any psychiatric disorder in the past 12 months is 15% for African Americans, 9% for Asian Americans, 16% for Hispanics, and 21% for non-Hispanic whites (8). Although African Americans and Hispanics have a lower risk of lifetime prevalence of mental disorders than do whites (9), they have a higher risk of persistence (longer course of illness) (10) and disability from mental illness (2). Furthermore, despite similarities in the measured prevalence of mental illnesses, racial/ethnic minority populations are disproportionately represented in vulnerable populations, such as the homeless and incarcerated, whose responses are not typically recorded for community surveys (3).

Racial/ethnic minorities who meet criteria for a mental disorder diagnosis may be undiagnosed or misdiagnosed. African Americans with an affective disorder are more likely to be diagnosed with schizophrenia than are white patients (11). Similarly, Hispanics are more likely to be diagnosed with affective disorders than are whites, although the difference is attenuated by adjusting for sociodemographic, care setting, and payment characteristics (12). Another issue of concern is the quality of care received by racial/ethnic minorities (13). African Americans and Hispanics are less likely than whites to receive guideline-based care for depression and anxiety (13), and African Americans are less likely than whites to be treated with atypical antipsychotic drugs and other newer agents (14).

Racial/ethnic minorities face barriers in accessing mental health care, particularly cost of care and fragmented services (3). Only 20% of American Indians report that they access care through the Indian Health Service (3). Furthermore, limited English proficiency and health literacy pose barriers for immigrant populations. In a 2007 study, only 8% of Hispanics who did not speak English, who reported a need for mental health services, received services. Likewise, of the non-English speaking Asian/Pacific Islander population who reported a need for mental health services, only 11% received services (15). Geographic accessibility is another barrier to care (2). People who live in rural areas have less access to mental health services than do their more urban counterparts (16). Even in rural areas where mental health services are available, some populations may not receive culturally appropriate services because of language barriers.

Medical insurance coverage is also a barrier to mental health care access among some racial/ethnic minorities. Sixteen percent of the overall US population is uninsured, compared with 25% of African Americans and 40% of Hispanics (3). Immigrants are 2.7 times more likely to be without health insurance than are US-born residents (17).

White and racial/ethnic minority populations have different sources of care. African Americans are more likely than whites to use emergency services, primary care physicians, and alternative treatments rather than specialists for diagnosis or treatment of mental disorders. African Americans are also overrepresented in inpatient treatment and underrepresented in outpatient treatment (3). A study of Southwest and Northern Plains tribal members living on or near reservations found that they are less likely than the overall population to seek help for mental illness (from specialists, other medical providers, or traditional or spiritual healers) (7). Asian Americans are less likely than whites to visit community mental health centers, emergency departments, self-help groups, or a psychiatric clinic in a general hospital (18).

## A Proposed Model to Overcome Disparities in Mental Health for Racial/Ethnic Minority Populations

We propose a model in which social determinants, interventions, and outcomes influence disparities in mental health and mental illness and are in turn subject to influence. The model illustrates the interactions of these factors to promote mental health and prevent mental illness (Figure).

**Figure 1:**
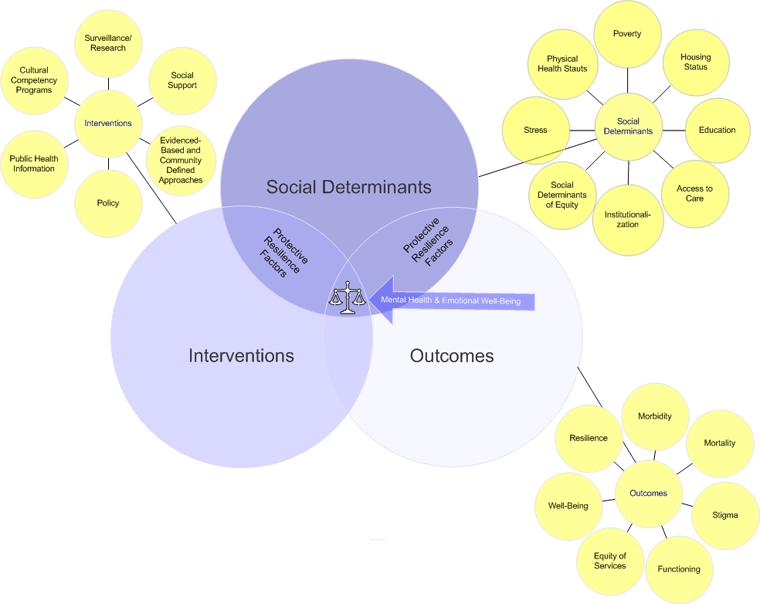
A proposed model to illustrate the interactions of social determinants, interventions, and outcomes to promote mental health.

### Social determinants

The model's social determinants are poverty, housing status, education, access to resources, institutionalization, social determinants of equity, stress, and physical health status. Living in poverty is associated with increased risk for mental, physical, and substance use disorders (19). Racial/ethnic minorities experience low socioeconomic status disproportionately in terms of income, occupation, and education, and this status has been significantly associated with mental illness (3).

Housing status refers to the effects of residential segregation in shaping socioeconomic status. Segregation in housing is associated with social problems such as high unemployment, reduced economic development, concentrated poverty, suboptimal education, and diminished access to health and mental health care (19). Segregated housing units often are substandard and expose occupants to high levels of environmental toxins. The high density of fast-food restaurants and scarcity of markets with fresh fruits and vegetables in these neighborhoods contribute to poor dietary habits (20). Furthermore, substandard housing and lack of infrastructure in poor urban neighborhoods where many racial/ethnic minority groups live have led to communities that are disenfranchised, with diminished social networks (21).

Racial/ethnic minorities are overrepresented among people who live in poverty, are homeless, or are institutionalized. Racial/ethnic minorities make up only 26% of the US population (1), but they make up 57% of the incarcerated population (22). Estimates from interviews with jail inmates in 2002 and with state and federal prisoners in 2004 revealed that more than half of all inmates have a recent history of mental illness (23); yet only 34% of state prisoners, 24% of federal prisoners, and 17% of local jail inmates have been treated for mental illness (23).

Social determinants of equity also influence mental health. For example, an intact family provides a strong, protective social network. Protective factors are defined as characteristics or conditions that diminish the likelihood that people will develop mental illness (5). Religious belief and social support are examples of protective factors. Although not conclusive, some research findings suggest a link between spirituality and well-being, which may operate through family ties (3). Negative social factors such as racism, racial bias, and discrimination contribute to poor physical and mental health among racial/ethnic minority populations (24). These populations also experience a higher rate of other negative life events such as victimization, abuse, and trauma, defined in the figure as stress (3).

The public health system must define disparities in diverse populations, set measurable goals for improvement, focus on community-based research, and acknowledge community needs pertaining to the social determinants of health.

### Interventions

Public health interventions are necessary to help prevent mental illness and promote mental health in racial/ethnic minority populations. The interventions depicted in our model are social support, surveillance/research, cultural competency programs, public health information, evidence-based and community-defined approaches, and policy.

Social support from the community, neighborhood, and family decreases emotional distress and the need for formal treatment (5). These informal networks can be seen as coping mechanisms that help make mental health programs successful (5). Current surveillance and research approaches are not adequate to address mental health disparities in racial/ethnic minority populations, particularly among Asian Americans and American Indians/Alaska Natives. Surveillance and research on these populations should be expanded to provide adequate data to monitor the prevalence, incidence, and severity of mental illness and access to care. These data may lead to the development of culturally based treatments and public health approaches to evaluate their efficacy (2). Language barriers and ignorance of cultural differences increase the likelihood of misdiagnosis. Including cultural competency programs in clinician training may help, as may encouraging racial/ethnic minorities to enter health care professions. Furthermore, dissemination of public health information through such techniques as social marketing could help eliminate the stigma of mental illness among some racial/ethnic minority groups and in so doing could encourage seeking help for mental illness. Public health approaches that are defined by the community, that are evidence-based (25), and that engage racial/ethnic minority populations are necessary to achieve desired outcomes. Finally, national policies must attempt to improve mental health among racial/ethnic minority populations through better access to both mental and physical health care systems (26).

### Outcomes

The outcomes in the model are morbidity, mortality, stigma, functioning, equity of services, well-being, and resilience. Higher morbidity and mortality are associated with chronic diseases among mentally ill people in some racial/ethnic minority populations (27,28). For example, among American Indians, a history of trauma, stress, or depression increases the risk for diabetes (29).

The stigma of mental illness among some racial/ethnic minority groups can cause people to avoid seeking specialty mental health care and refuse to receive medication or counseling (3). Such avoidance increases the likelihood of seeking care only at the stage of mental health crisis, when the risk of needing emergency services or involuntary hospitalization is higher. Other factors, such as lack of insurance, limited health literacy, and cultural beliefs, also interfere with help seeking, screening, and health assessment.

Positive outcomes include successful mental functioning, equity of services, and well-being, achieved through a combination of protective factors and tailored public health interventions. Resilience, or the capacity to recover from adversity, is often achieved through the positive effect of protective factors mediating negative social determinants. Resilience can preserve mental health even in the presence of poor socioeconomic circumstances. Religious participation, volunteerism, and neighborhood collaboration have also been identified as protective factors, strengthening resilience among racial/ethnic minority populations (3).

The intersection of social determinants of health, interventions, and outcomes illustrates how protective factors combined with interventions counterbalance potentially negative social determinants of mental health.

## Summary and Next Steps

For nearly a decade, federal reports have called for a public health approach that addresses the social determinants of mental illness to eliminate disparities in diagnosis and treatment. A successful approach must consider racial/ethnic minorities in their social contexts because these social determinants influence health outcomes.

As the US population grows increasingly diverse, providers must become more culturally sensitive in the treatment of mental illness. Provider training in the unique barriers to mental health among racial/ethnic minorities may be achieved by mandating such training in medical school and continuing education. Increases in the number of racial/ethnic minority health care providers could also increase the availability of culturally sensitive mental health care. However, the stigma of mental illness diagnosis and treatment among some groups may delay or prevent mental health care; social marketing campaigns may help alleviate such stigma.

To establish mental health in the public health arena, prevention should be emphasized. An example of universal prevention is the early identification of women with postpartum depression to reduce long-term risks for themselves and their infants (30). The need to recognize symptoms and provide early screening and treatment for common mental illnesses should be emphasized to racial/ethnic minority populations, the general public, and health care practitioners.

Collaborative efforts to eliminate health disparities are under way among several federal agencies. One example is the Federal Executive Steering Committee on Mental Health, an interagency group representing 9 federal agencies, led by the Department of Health and Human Services. This group was formed as a result of the New Freedom Commission on Mental Health (2), which called for a fundamental transformation of the US mental health care delivery system to one driven by consumer and family needs that focuses on building resilience, facilitating recovery, and eliminating disparities in mental health services. Among the steering committee's initial priorities are chronic disease and mental illness comorbidities.

We suggest the following strategies to address the disparities in mental health services and prevalence of mental illness in racial/ethnic minority populations:

- Surveillance systems should more comprehensively monitor the prevalence, severity, treatment access, and outcomes for mental illness. Surveillance programs are discussed elsewhere in this issue of *Preventing Chronic Disease* (31).
- Research priorities should include models that elucidate causal pathways for mental illness in vulnerable populations.
- Research should evaluate the effect of race, culture, language, mental illness, and chronic disease on illness and death and use the information to develop community engagement models.
- Programs and policies on mental health and illness should be integrated into the public health system.
- Federal agencies should collaborate to provide mental health care to incarcerated and recently released populations.
- Professional employee training, recruitment, and retention activities should lead to effective mechanisms for a multicultural mental health workforce.

A public health approach that promotes mental health among racial/ethnic minority populations and eliminates treatment disparities requires public policies that track and reduce disparities and seek solutions for these diverse communities. These goals will not be fulfilled quickly. They start with improving service programs, overcoming barriers to care, and building public health capacity.
